# Comparison between full face and hemifacial CBCT cephalograms in clinically symmetrical patients: a pilot study

**DOI:** 10.1590/2176-9451.20.2.083-089.oar

**Published:** 2015

**Authors:** Cintia Helena Zingaretti Junqueira, Guilherme Janson, Marisa Helena Zingaretti Junqueira, Lucas Marzullo Mendes, Eduardo Esberard Favilla, Daniela Gamba Garib

**Affiliations:** 1 Professor of Preventive Orthodontics, Centro de Estudos e Pesquisas, Rio de Janeiro, Rio de Janeiro, Brazil; 2 Full professor, Universidade de São Paulo (USP), College of Dentistry, Department of Orthodontics, Bauru, São Paulo, Brazil; 3 Chairman of the Postgraduate program in Preventive Orthodontics, Centro de Estudos e Pesquisas, Rio de Janeiro, Rio de Janeiro, Brazil; 4 Chairman of the Postgraduate program in Orthodontics, Faculdade do Centro Oeste Pinelli Henriques (FACOPH PR), Curitiba, Paraná, Brazil; 5 Head of the Service of Oral and Maxillofacial Surgery and Traumatology, Faculdade de Medicina de Petrópolis, Petrópolis, Rio de Janeiro, Brazil; 6 Full and associate professor, Universidade de São Paulo (USP), College of Dentistry, Department of Orthodontics, Bauru, São Paulo, Brazil

**Keywords:** Cone-beam computed tomography, Facial asymmetry, Diagnosis, Orthodontics

## Abstract

**INTRODUCTION:**

One of the advantages of cone-beam computed tomography (CBCT) is the possibility of obtaining images of conventional lateral cephalograms derived from partial or complete reconstruction of facial images.

**OBJECTIVE:**

This study aimed at comparing full face, right and left hemifacial CBCT cephalograms of orthodontic patients without clinical facial asymmetry.

**METHODS:**

The sample comprised nine clinically symmetrical patients who had pretreament full face CBCT. The CBCTs were reconstructed so as to obtain full face, right and left hemifacial cephalograms. Two observers, at two different times, obtained linear and angular measurements for the images using Dolphin 3D software. Dependent and independent t-tests were used to assess the reproducibility of measurements. Analysis of Variance and Kruskal-Wallis tests were used to compare the variables obtained in the CBCT derived cephalometric views.

**RESULTS:**

There was good reproducibility for CBCT scans and no statistically significant differences between measurements of full face, right and left hemifacial CBCT scans.

**CONCLUSIONS:**

Cephalometric measurements in full face, right and left hemifacial CBCT scans in clinically symmetrical patients are similar.

## Abstract (pt)

**INTRODUÇÃO:**

uma das vantagens da tomografia computadorizada de feixe cônico (TCFC) é a possibilidade de obtenção da imagem da telerradiografia em norma lateral convencional, por reconstruir parcial ou totalmente a face em uma visão sagital.

**OBJETIVO:**

o presente estudo teve como objetivo comparar as imagens de cefalogramas obtidas da face total e das hemifaces direita e esquerda por meio de TCFC, em pacientes sem assimetria clinica.

**MÉTODOS:**

a amostra foi composta por nove pacientes, clinicamente simétricos, que tinham em seus prontuários TCFC de face total. Os exames tomográficos foram reformatados para a obtenção de cefalogramas de face total e hemifaces direita e esquerda. Dois observadores, em dois momentos diferentes, obtiveram medidas lineares e angulares dessas imagens, utilizando o *software* Dolphin 3D. Testes *t* dependentes e independentes foram utilizados para verificar a reprodutibilidade das medidas nas imagens. Análise de variância (ANOVA) e o teste de Kruskal-Wallis foram utilizados para comparar as variáveis obtidas nas análises cefalométricas.

**RESULTADOS:**

houve boa reprodutibilidade para as imagens de TCFC e não houve diferença estatisticamente significante entre as medidas cefalométricas obtidas de cefalogramas de face total, hemifaces direita e esquerda.

**CONCLUSÕES:**

medidas cefalométricas obtidas de cefalogramas de face total, hemifaces direita e esquerda em pacientes clinicamente simétricos são semelhantes.

## INTRODUCTION

In the last decades, three-dimensional images have contributed to diagnosis in several fields, including Dentistry. Particularly Orthodontics can benefit from the advantages of cone-beam technology, a new type of computed tomography (CT) with a conic shape X-ray beam.^1^

Different from the traditional spiral CT, in which a fan-beam carries out several rotations around the patient, with cone-beam computed tomography (CBCT) a single rotation of x-rays and a solid panel sensor around the patient complete the exam. CBCT radiation dose is remarkably lower than spiral CT.^2^ It corresponds approximately to the effective dose generated for a panoramic, lateral x-ray and full-mouth periapical radiograph combined.^2^^,^ 4

In the CBCT exam, the field of view (FOV) can be adjusted to scan small or large areas, such as local impacted teeth and surrounding structures, or a complete face, in cases of initial diagnosis and treatment planning. Besides generating slices in all three planes of space, CBCT has the possibility to reconstruct two-dimensional images, such as panoramic or lateral cephalometric radiographs. The new technology leaves behind most of conventional x-ray disadvantages, including distortion, magnification and superimposition.^5^

Previous studies validated CBCT cephalogram images for two-dimensional dentofacial evaluation.^6^^,^ 8 Kumar et al,^6^ aiming to compare conventional lateral and CBCT cephalograms of ten dry skulls, found that CBCT reproduced conventional cephalometric radiographs with similar precision and accuracy. The same authors reproduced the study in 31 patients, in which linear and angular measurements were not statistically different for either one of the methods, except for the Frankfort mandibular plane angle.^7^ Cattaneo et al^9^ also compared conventional cephalometric radiographs with CBCT-synthesized cephalograms of 34 patients, concluding that CBCT can be successfully used to perform cephalometric analysis. Van Vlijmen et al^10^ compared linear and angular cephalometric measurements obtained from conventional and CBCT-synthesized cephalograms and found higher reliability for CBCT measurements and did not find significant differences between the two types of images. Chien et al^8^ compared the reliability of landmark identification in conventional cephalometric radiographs and CBCT 3D derived images and found lower intraobserver reliability for two-dimensional than for three-dimensional images.

Diagnosis and treatment planning for asymmetric patients is a considerable challenge in Orthodontics. Many studies have emphasized the applicability of 3D CT scans in these patients, with some authors recommending their own 3D cephalometric analyses.^11^^,^ 14 Measuring face asymmetries using two-dimensional cephalograms could be possible instead of using 3D reconstructions, which is more complex than the usual 2D cephalometry.

An important advantage of CBCT-derived cephalograms is the possibility to separately reconstruct the right and left sides of the face. Comparing one side to the other could bring relevant information about asymmetries location and size. This comparison is valuable, since eventual differences between both sides are not expected from symmetric patients.

No previous study compared the left and right side by means of CBCT-reconstructed cephalograms to assure whether there is equivalence of both sides in clinically symmetric patients. For this reason, the main purpose of this pilot study was to compare right and left CBCT-derived cephalograms of clinically symmetric orthodontic patients with full face CBCT-derived cephalograms.

## MATERIAL AND METHODS

This study was approved by the Institutional Review Board of Universidade Veiga de Almeida under protocol #157/09. The sample comprised nine orthodontic patients (six females and three males) with mean age of 37.5 years. Selection criteria included: absence of clinically relevant facial asymmetry, CBCT scan as part of initial orthodontic records and age greater than sixteen years old. All nine patients had sought orthodontic treatment exclusively due to dental malocclusion.

**Figure 1 -:**
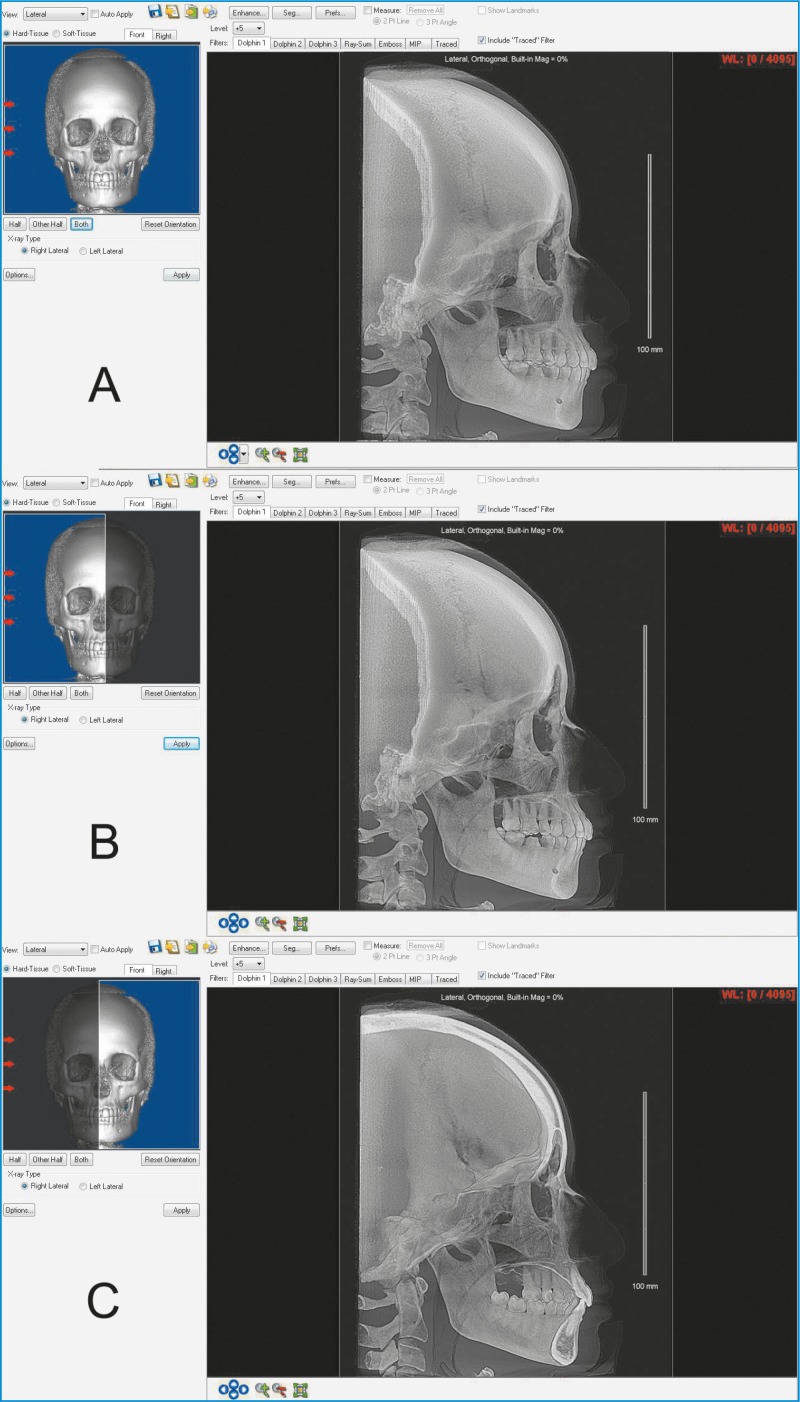
Different modalities of CBCT-derived cephalograms from the same subject A) Full face cephalogram. B) Right hemifacial cephalogram. C) Left hemifacial cephalogram.

All CBCT scans were acquired on an iCAT Cone-Beam 3-D System (Imaging Sciences International, LLC, Hatfield, Penn., USA) using a field of view of 22 cm (extended protocol) and voxel size of 0.4 mm. Using Dolphin 3D software (Dolphin Imaging and Management Solutions, Chatsworth, CA, USA), the CBCT scan of each patient was reconstructed to obtain three different images: a conventional cephalogram including the complete width of the face (Fig 1A); a lateral cephalogram, including only the right side of the face (Fig 1B); and a lateral cephalogram, including only the left side of the face (Fig 1C). Maximum intensity of projection (MIP) was selected for CBCT image visualization. In order to include all midsagittal structures, the reference used to limit hemifacial cephalogram reconstructions corresponded to the incisal edge midpoint of the maxillary central incisor of the opposite side. No patient had expressive maxillary midline deviation. Images were de-identified before evaluation.

Eleven cephalometric measurements were obtained on the cephalograms by means of Dolphin 3D software (Table 1). Measurements were performed by two previously calibrated examiners, in two different moments, within a two-week interval. After landmark identification, the software automatically measured all variables.

**Table 1 -:** Cephalometric variables analyzed.

Cephalometric measurements	Definitions
Linear measurements (mm)	
Maxillary length (Co-A)	Distance between Co and A
Anterior facial height (ANS-Me)	Distance between NS and Me
Mandibular length (Co-Gn)	Distance between Co and Gn
Cranial base (SN)	Distance between S and N
Angular measurements (degrees)	
ANB	Angle formed by landmarks A, N and B
SNA	Angle formed by landmarks S, N and A
SNB	Angle formed by landmarks S, N and B
SNGoGn	Angle formed by S-N line and GoGn plane
FMA	Angle formed by Frankfort and GoMe planes
1.PP	Angle formed by the long axis of the most anterior maxillary central incisor and the palatal plane
IMPA	Angle formed by the long axis of the most anterior mandibular central incisor and the GoMe plane

## Error of the method

Intra and interexaminer reproducibility of CBCT cephalograms was tested with dependent and independent t-tests, respectively, using the values obtained for all three different modalities of cephalograms. Intraexaminer error was calculated using the first and second values obtained by the examiners. As for interexaminer error analysis, only the first measurements of each examiner were used.

## Statistical analyses

Data normality was checked by Shapiro-Wilk test. Variables SN, ANB, SNA, SNB, FMA and 1.PP showed normal distribution, thus, intergroup comparison was performed with Analysis of Variance. Variables Co-A, ANS-Me, Co-Gn, SNGoGn and IMPA did not show normal distribution, thus, intergroup comparison was performed with Kruskal-Wallis test. Data used for analysis were those obtained by examiner 1, at time point 1. Results were considered statistically significant at *P < *0.05. All statistical tests were performed with SigmaPlot version 12.0 software (Systat Software, Inc. San Jose, California, USA).

## RESULTS

There were two systematic errors for examiner 1 and four for examiner 2 (Table 2). There was only one interexaminer error (Table 3). Six out of the eleven variables showed casual errors lower than 1 mm or 1.5 ^o^. Only two variables presented errors greater than 2 mm or 2^o ^(Tables 2 and 3).

**Table 2 -:** Paired t-test for intraexaminer error.

Cephalometric measurement	T_1_		T_2_		p	Dalhberg
	(Mean ± SD)		(Mean ± SD)			
Examiner 1						
Co-A	84.5 ± 6.6		84.5 ± 6.6		0.937	0.50
ANS-Me	68.1 ± 6.6		68.2 ±6.5		0.298	0.58
Co-Gn	113.9 ± 8.2		113.7 ± 7.7		0.354	0.94
SN	65.9 ± 3.7		66.0 ± 3.6		0.625	0.52
ANB	2.9 ± 2.6		2.7 ± 2.6		0.150	0.44
SNA	83.7 ± 3.5		83.5 ± 4.0		0.609	1.12
SNB	80.8 ± 2.7		80.8 ± 3.5		0.990	1.03
SNGoGn	31.6 ± 4.3		32.6 ± 5.8		0.036*	1.82
FMA	25.5 ± 4.4		26.5 ± 4.3		0.007*	1.40
1.PP	110.9 ± 8.0		111.3 ± 8.2		0.411	1.46
IMPA	91.0 ± 9.8		90.3 ± 9.4		0.227	2.13
Examiner 2						
Co-A	84.6 ± 6.4		86.5 ± 5.9		0.041*	0.99
ANS-Me	67.2 ± 6.4		68.0 ± 6.0		0.131	0.33
Co-Gn	114.1 ± 8.2		116.7 ± 7.4		0.029*	0.79
S-N	65.8 ± 4.0		66.4 ± 3.5		0.467	1.80
ANB	3.1 ± 2.9		3.2 ± 2.9		0.850	0.44
SNA	82.8 ± 3.9		82.7 ± 3.9		0.750	0.98
SNB	79.7 ± 3.3		79.5 ± 3.5		0.534	0.85
SNGoGn	34.4 ± 5.7		33.5 ± 5.6		0.044*	1.12
FMA	27.0 ± 4.3		26.1 ± 4.2		0.072	0.72
1.PP	112.5 ± 8.6		111.0 ± 8.4		0.041*	1.44
IMPA	91.2 ± 8.8		89.9 ± 9.1		0.050	2.08

* Statistically significant at P < 0.05.

**Table 3 -:** Independent t-test for interexaminer error (T1).

Cephalometric measurement	Examiner 1	Examiner 2	P value	Dalhberg
	Mean ± SD	Mean ± SD		
Co-A	84.5 ± 6.6	84.6 ± 6.4	0.951	0.53
ANS-Me	68.1 ± 1.2	67.2 ± 1.2	0.607	0.89
Co-Gn	113.9 ± 8.2	114.1 ± 8.2	0.928	0.79
S-N	65.9 ± 3.7	65.8 ± 4.0	0.912	1.20
ANB	2.9 ± 2.6	3.1 ± 2.9	0.727	0.52
SNA	83.7 ± 3.5	82.8 ± 3.9	0.390	1.31
SNB	80.8 ± 2.7	79.7 ± 3.3	0.183	1.48
SNGoGn	31.6 ± 4.3	34.4 ± 5.7	0.046*	2.63
FMA	25.5 ± 4.4	27.0 ± 4.3	0.237	1.57
1.PP	111.3 ± 8.2	112.5 ± 8.6	0.601	1.95
IMPA	91.0 ± 9.8	91.2 ± 8.8	0.947	2.35

* Statistically significant at P < 0.05.

No significant differences were observed among the three modalities of CBCT-synthesized cephalograms (Table 4).

**Table 4 -:** Comparison of the three image modalities (Analysis of Variance and Kruskal-Wallis tests).

Cephalometric measurement	Total face		Right hemiface		Left hemiface		P value
	Mean ± SD	Median	Mean ± SD	Median	Mean ± SD	Median	
Co-A*	84.72 ± 6.77	83.10	84.37 ± 7.03	82.30	84.48 ± 7.08	82.80	0.927
ANS-Me*	68.16 ± 6.84	68.40	68.14 ± 6.87	68.10	68.08 ± 7.11	68.60	0.996
Co-Gn*	114.29 ± 8.41	112.20	114.18 ± 8.51	111.60	113.48 ± 8.91	111.90	0.927
S-N	65.94 ± 3.80	65.30	65.72 ± 4.17	65.20	66.26 ± 3.65	65.90	0.958
ANB	2.77 ± 2.76	3.00	2.90 ± 2.67	3.00	3.06 ± 2.98	3.50	0.976
SNA	83.60 ± 4.31	82.40	83.98 ± 3.39	83.40	83.64 ± 3.19	83.10	0.972
SNB	80.82 ± 2.91	80.80	81.09 ± 3.09	81.10	80.59 ± 2.48	80.80	0.933
SNGoGn*	31.48 ± 4.70	32.00	31.12 ± 4.08	29.60	32.22 ± 4.74	30.60	0.755
FMA	25.67 ± 4.46	24.90	25.48 ± 4.45	25.80	25.56 ± 5.09	25.80	0.996
1.PP	111.31 ± 7.88	111.40	111.17 ± 8.82	112.70	111.50 ± 9.05	112.50	0.997
IMPA*	91.53 ± 9.06	95.10	90.29 ± 9.66	95.90	91.30 ± 11.65	92.00	0.973

* Kruskal-Wallis test.

## DISCUSSION

## Sample

This study was conducted based on a sample of nine orthodontic patients. This unpretentious number could be considered quite small to be representative. Sample reduced size is justifiable for a pilot study and was due to the inclusion criteria regarding absence of clinical asymmetry and post adolescent age.

Nevertheless, considering the radiation dose involved in computed tomography, it is not so easy to reach a large number of patients whose conditions justify submission to the exam. Nowadays, the ethic aspects related to researches including radiation are a delicate topic of debate. To be included in this study, in addition to having a CBCT scan, the patient could not have any clinically significant asymmetry.

It is important to emphasize that CBCT exposes the patient to a greater radiation dose compared to conventional radiographs.^3^ For this reason, currently CBCT should only be indicated when the benefits for a better diagnosis are greater than the individual detriment that radiation exposure might cause (ALARA principle).^5^ On the other hand, exposure to radiation in a CBCT exam is much lower than a conventional CT.^3^ In some specific situations, when it is necessary to assess details on bone structures, the CBCT technology is an alternative to replace its predecessor.

All individuals included in the present sample had a CBCT scan included in the initial orthodontic records for clinical reasons. Furthermore, the cephalograms generated helped to elaborate their treatment plan, along with dental cast and facial analysis. If a CBCT is included in the initial orthodontic records due to specific indication, it can be used to generate 2D images. Conventional cephalogram and panoramic radiograph may be eliminated from orthodontic records, thereby considerably reducing the radiation dose.

## Reproducibility of CBCT-derived cephalograms

Among the 11 cephalometric variables tested, only two showed intraexaminer systematic error for the first examiner and four for the second examiner (Table 2). Only one variable (SNGoGn) showed significant systematic interexaminer error (Table 3). These results evince the high reproducibility of cephalometric measurements in all modalities of CBCT cephalograms.

Some reproducibility error was expected because in cephalometry there are inherent errors involved.^15^ Although a two-week interval existed between the two measurements, randomly or systematically, two examiners will unlikely choose, at two different moments, exactly the same point, especially those of subjective landmarks. Some examples are the landmarks Gonion and Gnathion, which are difficult to identify, and may have contributed to the significant errors that appeared in SNGoGn, FMA, IMPA and Co-Gn. Gonion and Gnathion points were often involved in systematic and casual errors. Previous studies also reported higher values for errors of measurements involving these points.^16,17^

The SNGoGn angle was involved four times in errors of reproducibility (Tables 2 and 3). Some arguments may explain these results. Previous studies showed that the fronto-nasal suture (point N) can be easily identified in CBCT scans, whereas point Sella (point S) showed lower reproducibility.^8,9^ Sella is identified as a geometrical center of a circular structure with the same name in the center of the sphenoid bone. Additionally, temporal bone density can slightly obstruct clear visualization of midsagittal structures in MIP reconstructions of CBCT scans, since structures with higher density can hide structures of lower density.^9^

Random errors were observed for interexaminer comparisons. Even if previously calibrated, two different orthodontists examiners will hardly elect the exact same position for a certain landmark. All but one occurrence of interexaminer errors appeared in angular measurements. Furthermore, those were all angles formed by not only three, but four points. Including one more landmark identification process each and the inexorable imprecision, they were more likely to show unavoidable casual errors. Moreover, the involvement of dental measurements, such as 1.PP and IMPA in errors, is in agreement with previous studies.^17,18^

The high reproducibility found for CBCT cephalometric measurements in this study is in accordance with previous studies.^8,9,19,20^ Cattaneo et al^9^ found higher reproducibility for CBCT cephalograms compared to conventional cephalometric images in a sample of 20 patients, except for measurements NS-Ar and NS-Ba which also depend on the correct identification of point S. Chien et al^8^ compared intra and interexaminer reproducibility for 27 measurements obtained by six observers from ten conventional 2D cephalograms and their respective CBCT-derived 3D images. The authors concluded that the 3D images had improved reliability in certain landmarks* in vivo* when compared with two-dimensional images. Ludlow et al^20^ compared 24 landmarks identified by five observers during two separate sections and in conventional and CBCT-derived cephalograms. CBCT scans provided more precise identification. The authors reported that greater variability of some points in the mediolateral direction was probably related to inadequate definition of landmarks in third dimension. Chang et al,^19^ comparing the identification of 20 lateral cephalometric landmarks by 11 observers at two time points, concluded that the errors on CBCT-derived cephalograms were comparable to those on conventional digital cephalograms, and also that the Ba point was more reliable on CBCT-derived cephalograms.

## Comparison between full face and hemifacial CBCT scans

There was no significant difference for any cephalometric variable in the comparison between full face, right and left hemifacial CBCT scans (Table 4). Considering the absence of relevant clinical facial asymmetries in the sample, these results were expected.

No previous study compared the right and left side of CBCT-derived cephalograms. Therefore, according to the present results, right or left hemifacial CBCT cephalograms can be used for two-dimensional cephalometry in symmetrical patients with the advantage of a clearer identification of bilateral structures. Unlike conventional cephalometric radiographs, CBCT-reformatted images have no magnification or distortions in the orthogonal plane.^5^

One of the indications of 3D cephalometry is the assessment of patients with facial asymmetry.^21^^,^ 25 To locate and quantify facial asymmetry, in addition to using 3D reformatted CBCT scans^23,26,27 ^or multiplanar reconstructions,^21,28^ another option would be comparison of right and left CBCT-reformatted cephalograms, as previously performed with dry skulls.^28^ Because landmark location in three-dimensional images is more difficult and time-consuming, comparison between hemifacial CBCT cephalograms could be an alternative for clinical use.

Location of facial asymmetry represents an important factor influencing individual attractiveness.^29^ A comparison between unilateral cleft lip and palate, orthognatic Class III and Class I malocclusion individuals regarding attractiveness was performed. Although there were no differences in facial asymmetry between cleft and orthognathic surgery patients, the first group was rated as significantly less attractive. This result shows that not only the amount of asymmetry influences attractiveness, but also its location.^29^

An accurate exam to assess morphology and facial asymmetry, leading to successful treatment plans, including orthognathic surgery, is important. Mandibular asymmetries, such as chin deviation in Class III malocclusion patients, were examined by means of computed tomography revealing that they were due to greater growth and mesial inclination of the ramus and greater maxillary vertical excess in the opposite side.^30^ The precise location of asymmetry is crucial to determine details of surgical treatment planning.

## CONCLUSIONS

Cephalometric measurements in CBCT-derived cephalograms showed good reproducibility.

Cephalometric measurements in full face, right and left hemifacial CBCT scans, in clinically symmetrical patients, were similar.
